# An Advanced Lens Measurement Approach (ALMA) in post refractive surgery IOL power calculation with unknown preoperative parameters

**DOI:** 10.1371/journal.pone.0237990

**Published:** 2020-08-25

**Authors:** Nicola Rosa, Ferdinando Cione, Angela Pepe, Salvatore Musto, Maddalena De Bernardo

**Affiliations:** Department of Medicine, Surgery and Dentistry “Scuola Medica Salernitana”, University of Salerno, Salerno, Italy; Nicolaus Copernicus University, POLAND

## Abstract

**Purpose:**

To test a new method to calculate the Intraocular Lens (IOL) power, that combines R Factor and ALxK methods, that we called Advance Lens Measurement Approach (ALMA).

**Design:**

Retrospective, Comparative, Observational study.

**Setting:**

Department of Medicine and Surgery, University of Salerno, Italy.

**Methods:**

Ninety one eyes of 91 patients previously treated with Photorefractive Keratectomy (PRK) or Laser-Assisted in Situ Keratomileusis (LASIK) that underwent phacoemulsification and IOL implantation in the capsular bag were analyzed. For 68 eyes it was possible to zero out the Mean Errors (ME) for each formula and for selected IOL models, in order to eliminate the bias of the lens factor (A-Costant). Main outcome, measured in this study, was the median absolute error (MedAE) of the refraction prediction.

**Results:**

In the sample with ME zeroed (68 eyes) both R Factor and ALxK methods resulted in MedAE of 0.67 D. For R Factor 33 eyes (48.53%) reported a refractive error <0.5D, and 53 eyes (77.94%) reported a refractive error <1D, For ALxK method, 32 eyes (47.06%) reported a refractive error <0.5 D, and 53 eyes (77.94%) reported a refractive error <1 D. ALMA method, reported a MedAE of 0.55 D, and an higher number of patients with a refractive error <0.5 D (35 eyes, 51.47%), and with a refractive error <1 D (54 eyes, 79.41%).

**Conclusions:**

Based on the results obtained from this study, ALMA method can improve R Factor and ALxK methods. This improvement is confirmed both by zeroing the mean error and without zeroing it.

## Introduction

In case of previous refractive surgery, IOL power calculation is a great challenge for the ophthalmologist because of its epidemiological dimensions: according to the European Registry of Quality Outcomes for Cataract and Refractive Surgery (EUREQUO) data, reported by Manning et al. [1] the percentage of cataractous patients with previous refractive surgery shows a positive trend, going from 0.06% in 2008 to 0.22% in 2013, with an almost 4- fold increase in the number in just 6 years [1].

IOL power calculation after refractive surgery is not a simple task, and to improve the outcome, several formulas have been proposed [2–22]. To obtain patient's preoperative data is challenging, for this reason clinical history based methods are often unsuitable.

Among the methods that do not require the knowledge of the patient's clinical history, the first one, called R Factor, was published by Rosa et al. in 2002 [12].

The R Factor method provided satisfactory results but in some cases it produced an excessively myopic postoperative refraction [13,15]. Later, the same author developed a new method, identified as ALxK, that was supposed to be used with history based methods, when the patients’ clinical history was unknown [16,17]. Subsequently, comparing AL*K with the results obtained with R factor, a correlation between the post refractive surgery under-correction and the post cataract myopic error was found.

Based on this experience, a study aimed to test the combination of the previously described methods that was called Advanced Lens Measurement Approach (ALMA) was planned. To perform the study and to verify ALMA, the protocols published by Hoffer et al. were followed [23]. Since some studies suggest that zeroing out the mean error should not be done for atypical eyes [24], in this study, similarly to the study by Ma et al. [25] both zeroing out and not zeroing out the mean error, were performed.

## Material and methods

In this retrospective study, performed at the Salerno University Hospital, the data of 295 eyes of 295 patients that underwent cataract extraction and IOL implantation, following refractive surgery, were examined.

The study was performed according to the Declaration of Helsinki guidelines. A written informed consent was acquired from all the participants. This study was approved by the local Institutional Review Board, Cometico Campania Sud, Italy, (protocol. number 16544). None of the eyes in this study were used to develop either the R factor or the ALxK method.

Preoperative cataract surgery keratometry and axial length values were measured using the IOL Master 500 (Carl Zeiss Meditec, Dublin, CA) and patients’ postoperative refractions were obtained through both subjective and objective methods. Patients with a best corrected visual acuity less than 20/20, with moderate and severe dry eye, pterygium, eye surface diseases, unknown refraction after cataract extraction, unknown implanted IOL power, with refractive surgery different from myopia or refractive techniques other than PRK and LASIK were excluded from the study.

After applying the inclusion and exclusion criteria, 91 eyes of 91 patients were selected for the study (group A). This group presented the following parameters: keratometry 37.99 ± 2.59 D (median: 38.32 D), axial length 27.71 ± 2.03 mm (median: 27.50 mm).

The zeroing of the mean error (ME) was not achievable for all the selected eyes, because in some of them the implanted IOL constant but not the model was known or the IOL models were implanted in less than 3 patients, making the zeroing unreliable. This benchmark resulted in the selection of a 68 eyes sample (group B) that was appropriate for zeroing out the mean error. Group B had these parameters: keratometry 37.71±2.50 D (median: 38.01 D), axial length 28.02±2.01 mm (median: 27.91 mm). Other characteristics of both Groups are reported in Table 1.

**Table 1:** IOL models object of the study (Group A and Group B).

IOL model	N° Patients	Group A	Group B
Abbot AAB 00 Sensar	1	×	
Alcon Acrysof MA 60 BM	5	×	×
Alcon Restor SA60D3	2	×	
Alcon SA60AT	3	×	×
Alcon SN 60 WF	4	×	×
AMO Sensar AR 40e	14	×	×
AMO Tecnis 1 ZCB00	4	×	×
AMO Tecnis ZMA00	2	×	
AMO Tecnis Z9000	16	×	×
B&L Akreos Adapt	11	×	×
B&L Akreos AO MI60	2	×	
Corneal ACR 600 SE	2	×	
Corneal PHACNS 5	1	×	
Corneal Quatrix	1	×	
Curamed SA 60CZ	3	×	×
Hexavision HQ 203 HEP	2	×	
Hoya 118,5 AF1FY60AD	3	×	×
Hoya VA 60 BB	6	×	×
Soleko Fil611	1	×	
Zeiss CT Spheris 203	1	×	
Unknown	7	×	

Because ME error reflects the systematic bias of a method, checking its difference from zero was performed before zeroing it out [23].

To zero out the mean error, the Excel software (Microsoft Corporation) was utilized and the following steps were performed:

- Insertion of patient data (axial length, keratometry, A—constant, model and refractive power of implanted IOL, refraction after cataract extraction);
- Based on the these data, IOL powers were calculated using the SRK / T formula by applying the correction factors obtained both from the R Factor and ALxK formulas;
- The real refractive error was calculated for each patient, taking into account the refractive errors predicted with the implanted IOL according to both R factor and ALxK methods, and the effective one;
- The obtained data were subdivided In groups according to the IOL models and the applied formulas, the refractive errors were averaged and zeroed out by applying the “Goal seek” option for the "What if analysis" function in Excel [23].

In both A and B groups, the following absolute values were calculated for both R factor and ALxK methods:

- Median error;
- Mean error;
- Number of patients with refractive error <0.5 D and <1.0 D;
- Percentage of patients with refractive error <0.5 D and <1.0 D;
- Minimum, maximum and standard error;
- 95% confidence interval around the mean value.

Moreover, the parameter AL*K, where AL = axial length and K = mean keratometry value, was identified. Each group was then divided into two subpopulations, based on the AL*K value:

- Subpopulation with AL*K value > 1060 (groups A1 and B1), that should have been undercorrected after refractive surgery [16];
- Subpopulation with AL*K value <1060 (groups A2 and B2), that should have been fully corrected by refractive surgery [16].

ALMA method was obtained combining R Factor results when AL*K <1060 and the ALxK results when AL*K> 1060. ALMA, R Factor and ALxK formulas in A and B groups were compared.

Descriptive statistics, performed with the Excel software (Microsoft Corporation), were used to describe population’s characteristics and IOL power calculation’s accuracy. Statistical analysis was performed with SPSS 23.0 (SPSS, Inc., Chicago, IL). The normality of data was examined by the Kolmogorov-Smirnov test before zeroing out the mean error. For screening whether the ME was significantly different from zero, one-sample T-test or Wilcoxon-signed-rank test were used. The Wilcoxon-signed-rank test was performed to compare the median absolute errors of the different methods analyzed in group A. Bootstrapped estimates were applied to perform T tests and confidence intervals within group B, as per Hoffer et al. [26]. Bootstrapped estimates were preferred to non-parametric test because when transforming the data the older methods established on ranks tend to be underpowered; they tend to be less likely to detect a statistically significant difference, with the high risk running into a type II error [26]. A P value of less than 0.05 was considered statistically significant.

## Results

All data were normally distributed (P > 0.05), except for refractive errors obtained with R factor method in Group A (P = 0.012). To check whether MEs obtained from R Factor and ALxK methods in Group B and ALxK method in group A were significantly different form zero, one-sample T test was performed. Whereas, to verify the same parameter for refractive errors obtained by R factor method in Group A, due to its abnormal distribution, Wilcoxon-signed-rank test was applied. In all the cases, MEs for all IOL power calculation methods were statistically different from zero (P < 0.001).

### Group A

In this group, to compare R Factor and ALxk results, the mean error was not zeroed out and the manufacturer’s suggested A-constant was utilized in IOL power calculation (Table 2).

**Table 2:** A—Constant before and after zeroing out the mean error for R factor and ALxK methods, when possible.

IOL model	Manufacturer’s suggested A—constant	R factor’s modified A—constant	ALxK’s modified A—constant
Abbot AAB 00 Sensar	118.9	X	X
Alcon Acrysof MA 60 BM	118.9	117.8	117.5
Alcon Restor SA60D3	118.1	X	X
Alcon SA60AT	118.4	116.8	117.0
Alcon SN 60 WF	118.7	118.3	118.3
AMO Sensar AR 40e	118.4	116.4	116.2
AMO Tecnis 1 ZCB00	118.8	115.0	116.5
AMO Tecnis ZMA00	119.1	X	X
AMO Tecnis Z9000	119.0	118.3	118.1
B&L Akreos Adapt	118.0	117.9	118.3
B&L Akreos AO MI60	118.4	X	X
Corneal ACR 600 SE	120.0	X	X
Corneal PHACNS 5	118.5	X	X
Corneal Quatrix	119.6	X	X
Curamed SA 60CZ	118.8	113.5	114.3
Hexavision HQ 203 HEP	118.2	X	X
Hoya 118,5 AF1FY60AD	118.4	117.2	118.1
Hoya VA 60 BB	118.7	116.1	115.7
Soleko Fil611	119.0	X	X
Zeiss CT Spheris 203	118.0	X	X

This group was subdivided into two subgroups: A1 when AL*K ≥ 1060 (38 eyes) and A2 when AL*K <1060 (53 eyes).

In A1, with the R factor method, 14 eyes (36.84%) reported a refractive error <0.5 D and 19 eyes (50.00%) reported a refractive error <1.0 D, with a median absolute error of 1.07 D. With the ALxK method, 17 eyes (44.74%) reported a refractive error <0.5 D, and 29 eyes (76.32%) reported a refractive error <1.0 D, with a median absolute error of 0.72 D. Therefore, in A1 it was observed that ALxK obtained significantly better results than R Factor (P = 0.003).

In A2, with the R factor method, 22 eyes (41.51%) reported a refractive error <0.5 D and 31 eyes (58.49%) reported a refractive error <1.0 D, with a median absolute error of 0.96 D. With the ALxK method, 14 eyes (26.42%) reported a refractive error <0.5 D, and 24 eyes (45.28%) reported a refractive error <1.0 D, with a median absolute error of 1.44 D. Therefore, in A2 it was observed that R Factor obtained significantly better results than ALxK method (P < 0.001).

**Fig 1:**
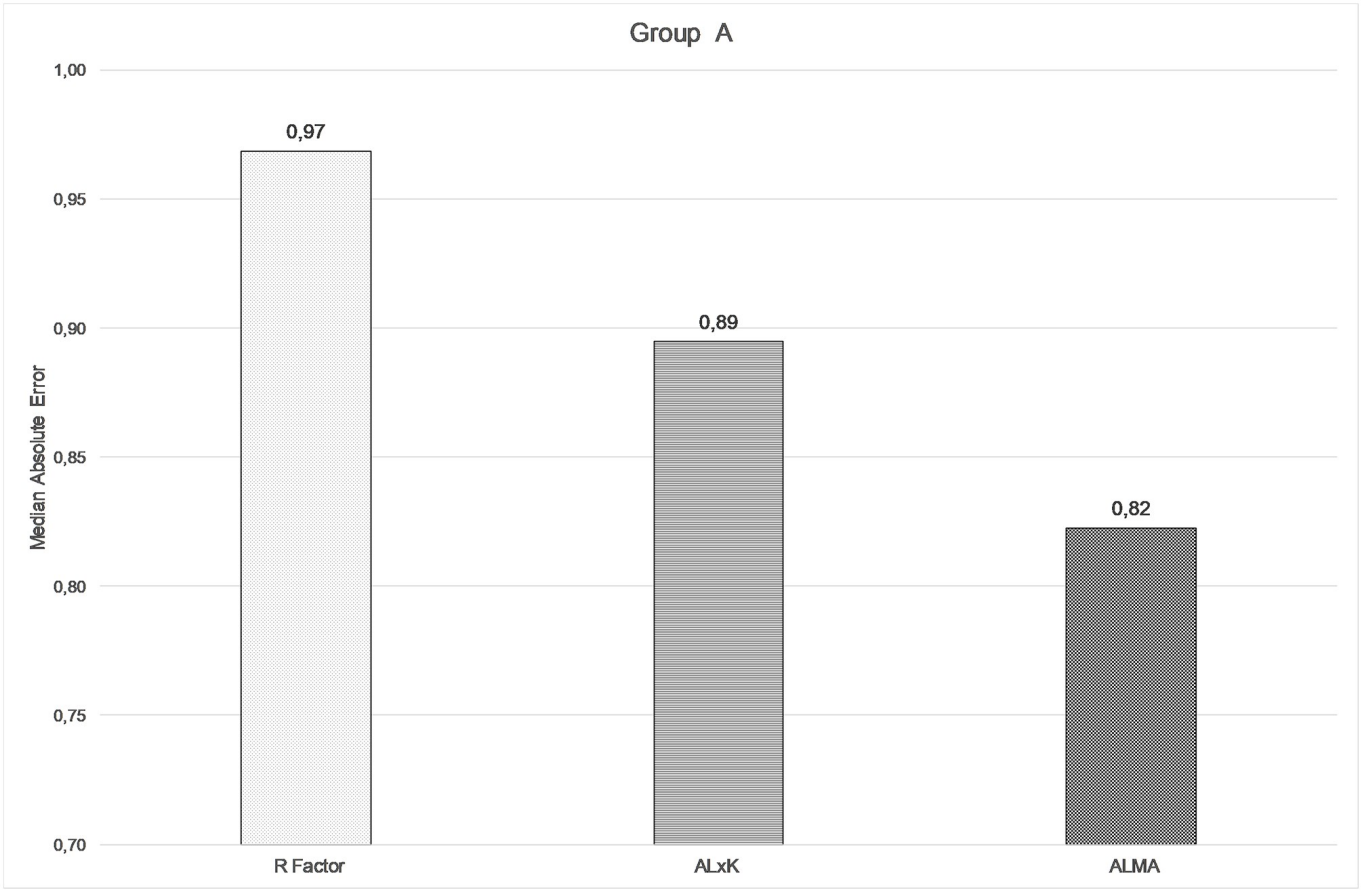
Comparison between median absolute errors by using R factor, ALxK, and ALMA methods in group A (91 patients, mean error not zeroed out).

**Fig 2:**
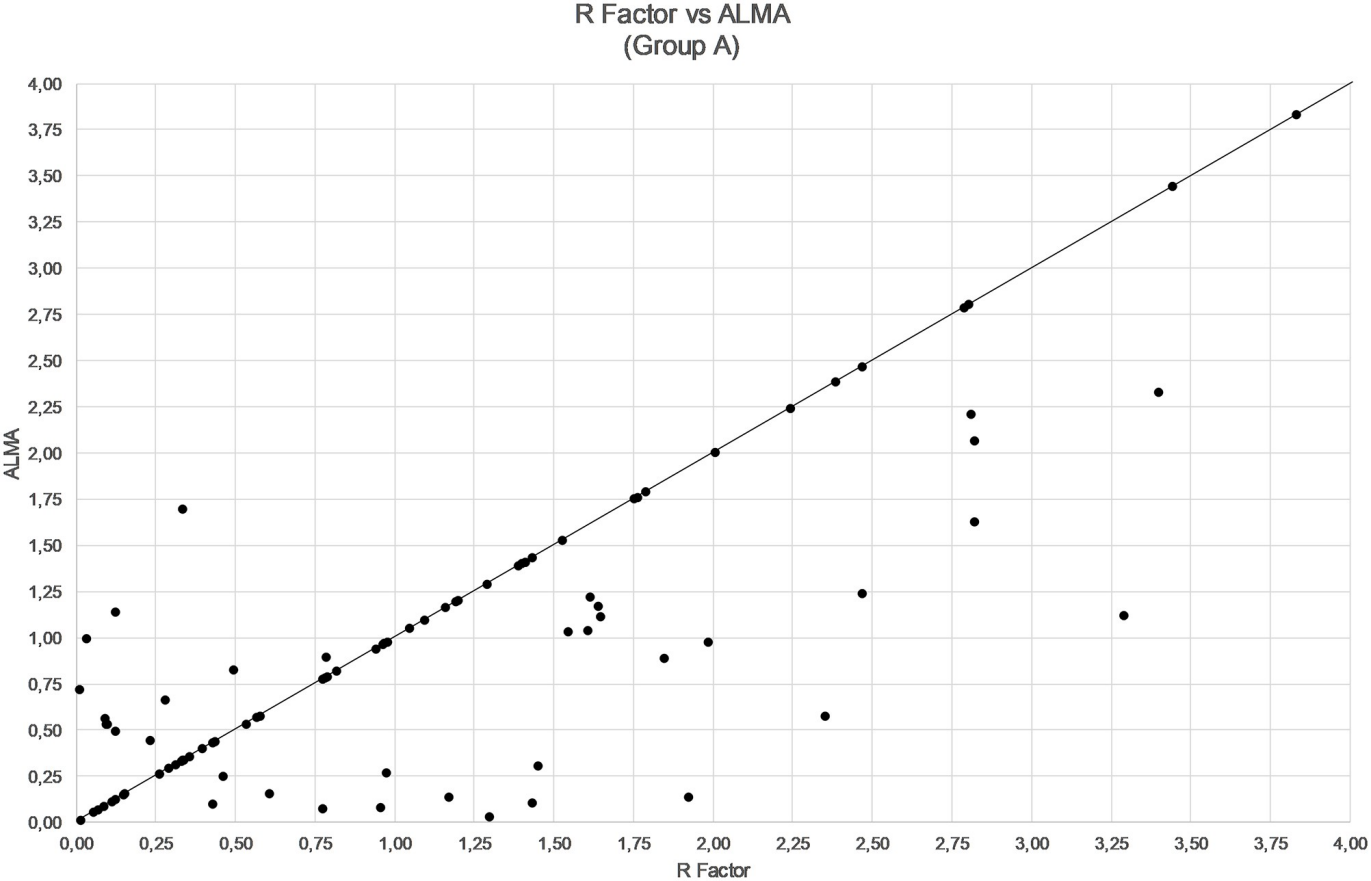
Comparison between refractive errors by using R factor and ALMA methods in group A (91 patients, mean error not zeroed out).

**Fig 3:**
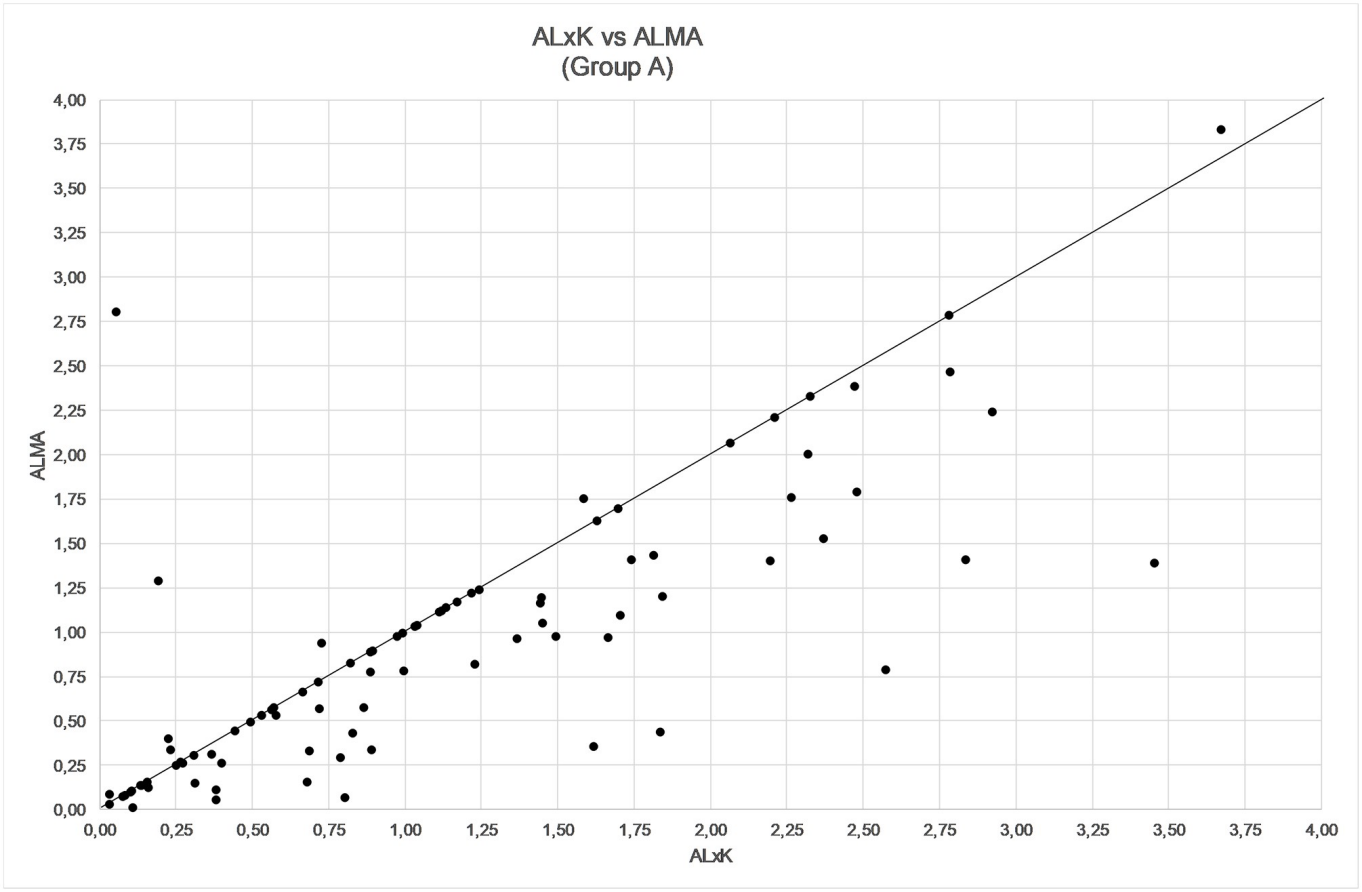
Comparison between refractive error by using ALxK and ALMA methods in group A (91 patients, mean error not zeroed out).

The comparison among ALMA, R factor and ALxK methods are shown in Figs 1–3 and in Table 3. From these results, ALMA appears to be superior to R Factor (P = 0.003) and ALxK (P < 0.001).

**Table 3:** Comparison between R Factor, ALxK and ALMA methods in Group A.

Group A			
Formula	R Factor	ALxK	ALMA
**<0.5 D**	36–39.56%	31–34.07%	39–42.86%
**<1.0 D**	50–54.95%	53–58.24%	60–65.93%
**Median absolute error**	0.97D	0.89D	0.82D
**MIN/MAX error**	0.01/4.39D	0.03/5.55D	0.01/4.39D
**STD error**	0.10 D	0.11 D	0.09 D
**95% confidence interval**	0.96–1.37D	0.99–1.43D	0.83–1.17D

### Group B

In this group, the mean error check showed that, for each method, it was different from zero (p < 0.001) so it was mandatory to zero it out. The modified lens constants obtained by zeroing out the mean error are shown in Table 2, and its effect is described in Table 4.

**Table 4:** Group B after zeroing out the Mean Error (ME).

*Formula*	*N° < 0*.*5 D*	*% <0*.*5 D*	*N° < 1*.*0 D*	*% <1*.*0 D*
*R factor*	29	42.65%	37	54.41%
*R factor (ME = 0)*	33	48.53%	53	77.94%
*ALxK*	23	33.82%	40	58.82%
*ALxK (ME = 0)*	32	47.06%	53	77.94%

This group was subdivided into two subgroups: B1 when AL*K ≥ 1060 (32 eyes) and B2 when AL*K <1060 (36 eyes) and the results are shown in Table 5.

**Table 5:** Characteristics, data and results for Group B1 (32 patients) and Group B2 (36 patients).

	Group B1 (AL*K>1060)		Group B2 (AL*K<1060)	
Formula	R Factor	ALxK	R Factor	ALxK
**Median absolute error**	0.52D	0.42D	0.71D	0.79D
**MIN/MAX error**	0.01/1.95D	0.12/2.52D	0.05/2.33D	0.03/2.08D
**STD error**	0.09D	0.10D	0.09D	0.09D
**95% confidence interval**	0.50–0.87D	0.47–0.83D	0.61–0.97D	0.67–0.99D

In B1 it was observed that ALxK obtained significantly better results than R Factor (P < 0.001).

In B2 it was observed that R Factor obtained significantly better results than ALxK method (P < 0.001).

**Fig 4:**
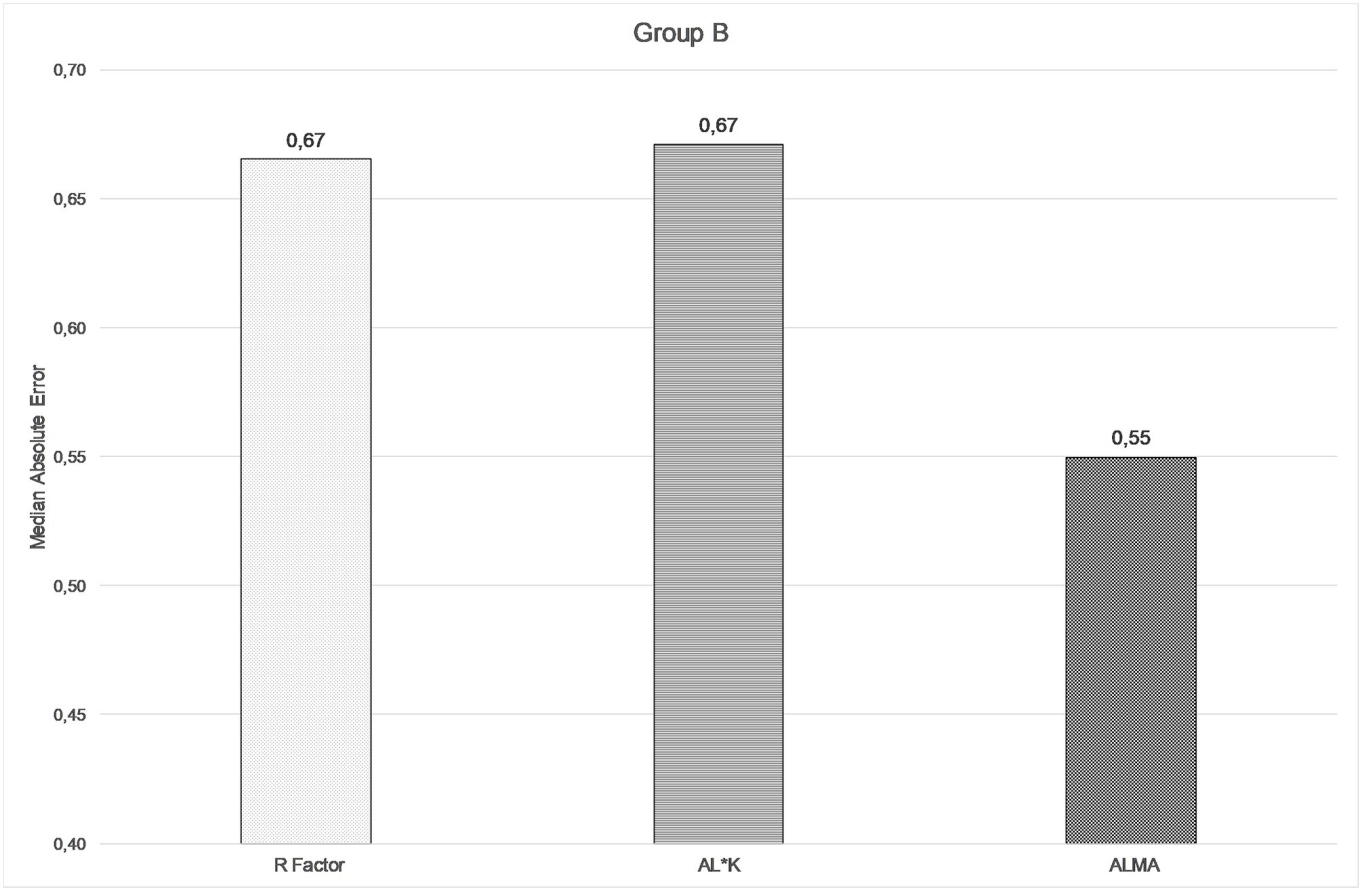
Comparison between median absolute errors by using R factor, ALxK, and ALMA methods in group B (68 patients, mean error zeroed out).

**Fig 5:**
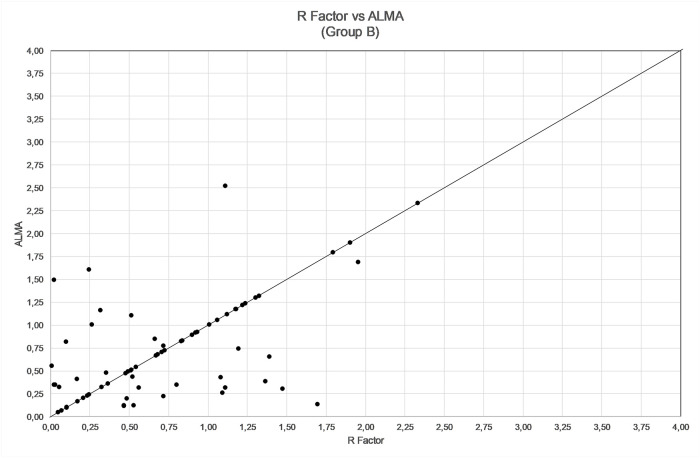
Comparison between refractive errors by using R factor and ALMA methods in group B (68 patients, mean error zeroed out).

**Fig 6:**
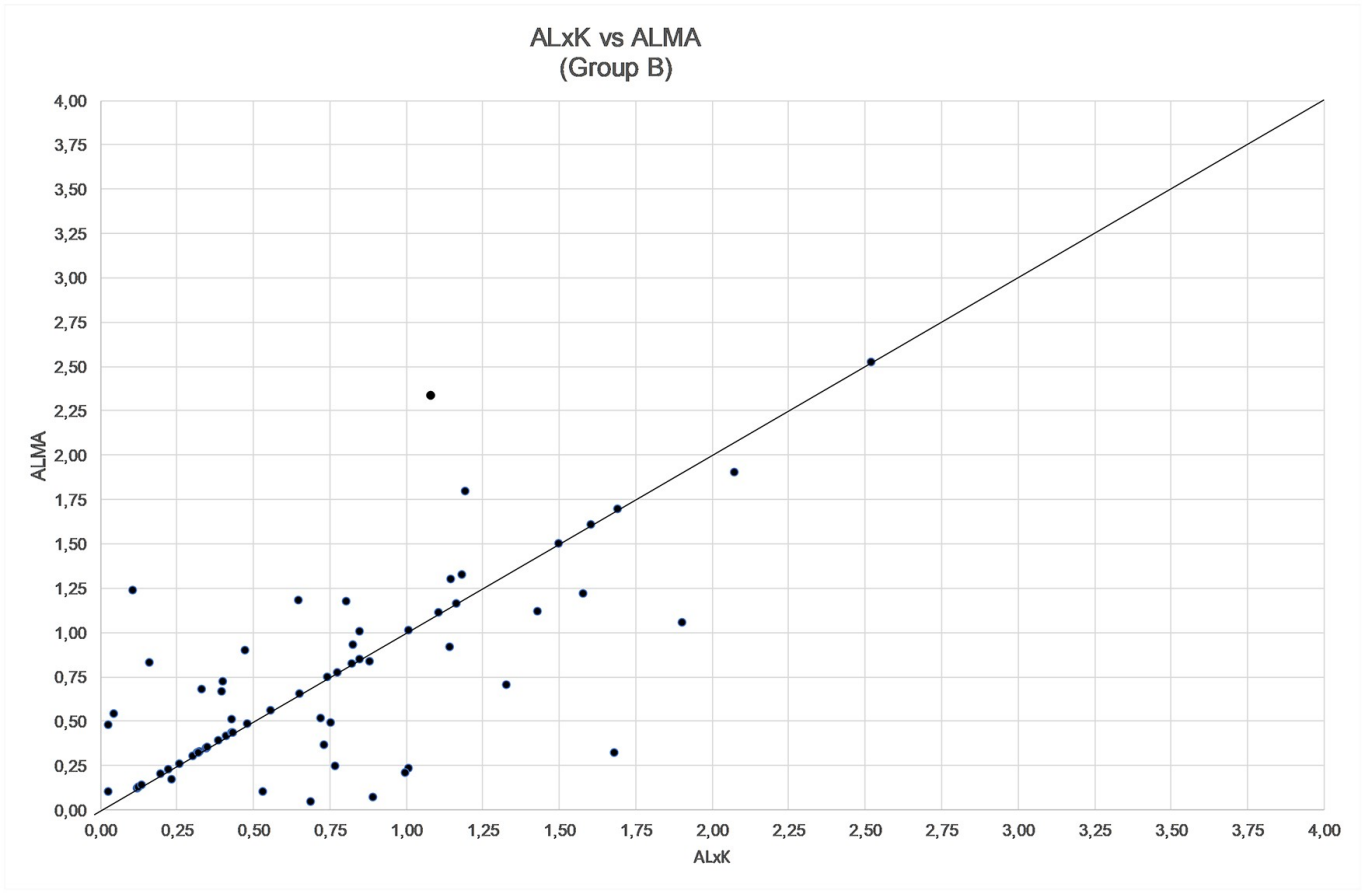
Comparison between refractive error by using ALxK and ALMA methods in group B (68 patients, mean error zeroed out).

The comparison among ALMA, R factor and ALxK methods are shown in Figs 4–6 and in Table 6. From these results, ALMA appears to be superior to both R Factor and ALxK (P < 0.001).

**Table 6:** Comparison between R Factor, ALxK and ALMA methods in Group B.

Group B			
Formula	R Factor	ALxK	ALMA
**% <0.5 D**	33–48.53%	32–47.06%	35–51.47%
**% <1.0 D**	53–77.94%	53–77.94%	54–79.41%
**Median absolute error**	0.67D	0.67D	0.55D
**MIN/MAX error**	0.03/2.52D	0.03/2.52D	0.05/2.52D
**STD error**	0.07D	0.07D	0.07D
**95% confidence interval**	0.61–0.86D	0.63–0.86D	0.59–0.85D

## Discussion

The post-operative refractive error, caused by an incorrect IOL power calculation, is the major complain after cataract surgery.

PRK and LASIK modify the corneal structure and geometry hence, the IOL power calculation after refractive surgery is more challenging due to:

- Inaccurate measurement of anterior keratometry;
- Keratometric index variation;
- Wrong effective lens position (ELP) estimation [27,28].

All these will result in a 14% to 25% IOL power calculation underestimation that can require the inappropriate IOL replacement [2–5].

To improve the IOL power calculation in patients with previous refractive surgery, several methods have been proposed [1,5]. These methods can be divided into two groups according to the need to know the patient’s clinical history, namely preoperative keratometry, preoperative refraction and achieved refraction after refractive surgery.

Holladay was the first to introduce a method based on the clinical history, followed by Hoffer [6]. Later on, several other papers, needing the clinical history, have been published in the international literature [7–11].

Nevertheless, it is not always possible to trace back to the patient's preoperative data. For this reasons, other authors tried to develop methods regardless of the patient's clinical history. The first of these methods, called R Factor, was published by Rosa et al. in 2002. This method utilizes a corneal radius correction factor which allows the hyperopic refractive error reduction [12].

Following this study, other methods that do not require the patient's clinical history have been published, and are among the most utilized [19–22].

Despite the advantages of the no clinical history methods, a gold standard method has not yet been found [19,29].

The R factor method, for example, in some cases has led to an excessive myopic post-surgical error [13,15]. Later, Rosa et al. developed a new method, identified as ALxK, that was supposed to be used with history based methods, when the patients’ clinical history was unknown [16,17]. They demonstrated that in patients that underwent refractive surgery, with results close to emmetropia, (± 0.5 D) the mean AL x K was 1005.91 ± 25.88. Thus, 95% (mean ± SD) were in the range of 954 and 1058, meaning that there was a 95% possibility that this range included patients with near full correction [16]. On this basis, the value of AL*K = 1060 was considered as the landmark. Subsequently, comparing AL*K with the results obtained with R factor, a correlation between the post refractive surgery under-correction and the post cataract myopic error was found. In this paper it was therefore assumed that above this value the ALxK method could have a better outcome than R factor, meanwhile below this value R factor method could have a better outcome than ALxK one. Two subpopulations, Group B1 and Group B2, based on the parameter AL*K, were identified from Group B to test this hypothesis. ALxK method gave a better refractive outcome than R factor one when the parameter AL*K > 1060. On the opposite, R factor method gave better refractive outcome than ALxK one when the parameter AL*K < 1060, as shown in Table 5.

The same procedure, performed for group A produced the same outcomes, as described above. These subgroups analysis, according to AL*K, confirmed the above-mentioned hypothesis and it was a necessary step to achieve ALMA approach.

This study achieves and proposes an advanced lens measurement approach (ALMA). This approach is a mixed theoretical–regression method, based on the SRK-T formula, structured as follows:

- Eyes with AL*K <1060—R Factor application;
- Eyes with AL*K> 1060—R factor modified according to ALxK.

Comparing the three methods, namely R factor, ALxK and ALMA to each other, ALMA method demonstrates significant advantages over two others, as shown in Figs 4–6, Table 6 (with zeroing out the mean error), and in Figs 1–3, Table 3 (without zeroing out the mean error). In both cases, with zeroing out and without zeroing out the mean error, P value was smaller than 0.05.

Considering the new scientific developments concerning IOL power calculation, it is essential to update and to try to improve the R factor accuracy [23,30]. Hence, the objective of this paper is to study and to test the use of the ALxK regression formula in improving the R factor results (ALMA).

In the last years several formulas to be used for the IOL power calculation after refractive surgery, have been proposed and compared. Unfortunately, up to 2015, no protocols indicating the common guidelines for testing the different methods accuracy were present in the literature and in none of them the bias of the chosen lens constant was considered [23].

This is a very important point because for clinical studies the independence from the lens constant is mandatory [23,30].

There are two ways to eliminate the systematic error: to optimize the lens constant for each formula in the study group and to reanalyze the outcomes or to zero out the mean error by adjusting the refractive prediction error for each method. The last option was suggested both by Hoffer et al. and Wang et al. [23,30] and it was chosen for this study.

Despite the criticism received by this protocol, the zeroing of the mean error has been recommended by other study groups too [30–32] and it represents a milestone in the realization of valid and accurate studies and the most recently published papers concerning IOL power calculation took it into consideration [25,33–40].

Unfortunately so far, most of the studies dealing with IOL power calculation with a and without previous refractive surgery did not consider the recommendations by Hoffer et al. [24,41–46]. The reasons for this are different, some authors did not apply the zeroing of the mean error just for technical difficulties [47].

Other authors considered inappropriate to zero out the mean error for atypical eyes, e.g. post refractive surgery eyes [24]. However, this limitation was not included in the recommendations published by Hoffer et al. [23]. In addition, Wang et al. described the possibility to optimize the lens constant for atypical eyes subset [30]. Furthermore, other studies that perform zeroing out of the mean error in atypical eyes, e.g. vitrectomized eyes [38], or short and long eyes [39] have been published.

Hoffer et al. suggested to avoid multiple IOLs in a study when reporting a method accuracy [24]. This is acceptable but obtaining a large database in most of the studies regarding IOL power calculation after refractive surgery is difficult, hence multiple IOL models were analyzed [22,25,41–44]. As reported by Abulafia et al. [48] more than one IOL model is appropriate when limited data are available.

On the other hand, the use of multiple IOL models can be considered acceptable when zeroing out the mean error is performed for each IOL model. This occurs to minimize the bias by the implementation of multiple IOL models, as per Hoffer protocols [23]. Similarly, recommendation published by Wang et al. do not prohibit the use of multiple IOL models [30].

Given the presence of the above mentioned debate, and following the example of other papers [25], this study was performed both by applying the zeroing out and without zeroing out the mean error, giving more strength to the paper.

The experimental design in this study provided some significant advantages over previous studies, but it has some limitations. Due to the small number of patients implanted with some IOL models it was only possible to zero out the mean error in 68 eyes of 95. Increasing the patients’ number would allow to identify the improvements by ALMA method. For example, in group B, there was hardly any difference in the number of patients (1 patient) with a refractive error <1 D between the R Factor and the ALMA methods, but in Group A, with an higher number of examined patients, this difference became considerable (10 patients). Considering that in clinical practice the lens constant is not optimized by zeroing out the mean error, the differences in the refractive outcomes observed in group A could be clinically more relevant, as shown in Table 3.

Besides, the low number of patients for some IOL models, could cause an alteration of the A-constant, due to an outlier. Hence, zeroing out the mean error could mute these outliers. However, the use of median absolute error instead of the mean error and the presence of multiple IOL models could limit the influence of eventual outliers.

Although the primary objective of this paper was to study and to verify if ALMA approach could be used to improve the R factor results, additional limitation relates to the inability to compare ALMA with other methods, such as Barrett True-K formula [22]. Unfortunately, this comparison was not possible because many other formulas are not published in the literature and hence zeroing out mean error to compare refractive errors was unreliable.

In conclusion ALMA method can be used to improve the results obtained by R Factor when the parameter AL*K>1060. This improvement is confirmed both with and without zeroing out the mean error.

Zeroing out of the mean error has been proposed in experimental practice, in order to compare the various IOL calculation formulas in retrospective studies, but it could be introduced in clinical practice to improve the IOL power calculation [23,30–32]. Further studies on a larger patients’ database are imperative to understand the feasibility of the proposed method for clinical practice.

## Supporting information

S1 Data The complete dataset of these patients have been included in the Excel file uploaded as a S1 Data. (XLSX)
